# Ankle dorsiflexion assistance of patients with foot drop using a powered ankle-foot orthosis to improve the gait asymmetry

**DOI:** 10.1186/s12984-023-01261-1

**Published:** 2023-10-20

**Authors:** Wonseok Shin, Dongwoo Nam, Bummo Ahn, Sangjoon J. Kim, Dong Yeon Lee, Suncheol Kwon, Jung Kim

**Affiliations:** 1 AI·Robotics R&D Group, Korea Institute of Industrial Technology, Ansan, 15588 Republic of Korea; 2 School of Korea Institute of Industry Technology, Robotics and Virtual Engineering, University of Science and Technology, Ansan, 15588 Republic of Korea; 3 Henry Samueli School of Engineering Department of Mechanical and Aerospace Engineering, University of California, Irvine, 92697 USA; 4 Department of Orthopedic Surgery, Seoul National University Hospital, Seoul, 03080 Republic of Korea; 5 Department of Mechanical Engineing, Korea Advanced Institute of Science and Technology (KAIST), Daejeon, 34141 Republic of Korea

**Keywords:** Foot drop, Ankle, Gait asymmetry, Dorsiflexion, Assistance

## Abstract

**Background:**

Foot drop is a neuromuscular disorder that causes abnormal gait patterns. This study developed a pneumatically powered ankle-foot orthosis (AFO) to improve the gait patterns of patients with foot drop. We hypothesized that providing unilateral ankle dorsiflexion assistance during the swing phase would improve the kinematics and spatiotemporal gait parameters of such patients. Accordingly, this study aims to examine the efficacy of the proposed assistance system using a strategy for joint kinematics and spatiotemporal gait parameters (stride length, swing velocity, and stance phase ratio). The analysis results are expected to provide knowledge for better design and control of AFOs in patients with foot drop.

**Method:**

Ten foot drop patients with hemiparesis (54.8 y ± 14.1 y) were fitted with a custom AFO with an adjustable calf brace and portable air compressor for ankle dorsiflexion assistance in the gait cycle during the swing phase. All subjects walked under two different conditions without extensive practice: (1) barefoot and (2) wearing a powered AFO. Under each condition, the patients walked back and forth on a 9-m track with ten laps of level ground under the supervision of licensed physical therapists. The lower-limb joint and trunk kinematics were acquired using 12 motion-capture cameras.

**Results:**

We found that kinematic asymmetry decreased in the three lower-limb joints after ankle dorsiflexion assistance during the swing phase. The average ankle-joint angle increased after using the AFO during the entire gait cycle. Similarly, the knee-joint angle showed a slight increase while using the AFO, leading to a significantly decreased standard deviation within patients. Conversely, the hip-joint angle showed no significant improvements with assistance. While several patients exhibited noticeably lower levels of asymmetry, no significant changes were observed in the average asymmetry of the swing velocity difference between the affected and unaffected sides while using the AFO.

**Conclusion:**

We experimentally validated that ankle dorsiflexion assistance during the swing phase temporarily improves gait asymmetry in foot-drop patients. The experimental results also prove the efficacy of the developed AFO for gait assistance in foot-drop patients.

## Introduction

Foot drop is a common symptom of brain or spinal cord disorders such as stroke, multiple sclerosis, and cerebral palsy [1]. These diseases typically lead to weaknesses or spasticity in the ankle dorsiflexor muscles that hinder the normal gait pattern. Accordingly, patients with foot drop experience two primary challenges: foot slap during the loading response and toe drag during the swing phase [2]. To compensate for these challenges, patients generally exhibit compensatory behaviors such as hip hiking and circumduction [3, 4].

Ankle-foot orthosis (AFO) has been used as a representative solution to improve gait quality by decreasing the ankle kinematic asymmetry in patients with foot drop [5, 6]. A leaf-spring-based AFO was developed to restrict ankle dorsiflexion by providing plantar flexion torque through a passive spring component [7, 8]. Several studies have reported that passive AFOs have a trade-off between the range of motion and power capacity of the system, depending on passive stiffness [9–11]. Blaya et al. [12] discovered that modulating the mechanical impedance of the AFO prevented slap foot occurrence and improved kinematic abnormalities, compared to zero- or constant-joint impedance modes. Shorter et al. [13] evaluated the kinematic efficacy of ankle dorsiflexion assistance using a portable AFO. Awad et al. [14] provided bidirectional assistance for both dorsiflexion (DF) and plantarflexion (PF) targeting patients with hemiplegic walking. Previous AFOs share similar designs and control schemes [15].

Adding mass to the legs, particularly directly to the ankle, impedes natural gait and hinders leg kinematics [16–18]. The Harvard exosuit that uses cable-based power transmission is the most representative example of a remote actuation system applied to assist patients with abnormal gait [19, 20] by minimizing the added mass to the ankle joint. The mass added to the ankle joint was sufficiently small (~ 0.5 kg) to avoid impeding the natural gait, resulting in a metabolic penalty. Pneumatic transmission is another example of remote force transmission [21–27]. A pneumatic-based wearable robot generally consists of a compressor as an energy source and a pneumatic actuator, connected through the air tube.

In our previous study, we developed an untethered AFO powered by a fully portable pneumatic compressor for foot drop correction [26, 27]. The custom portable compressor enabled a fully untethered system for patients with foot drop, which was impossible with most commercially available pneumatic pumps with excessive size and weight [26] based on a simple on-off valve controller. We evaluated the immediate efficacy of the developed pneumatic AFO for foot drop correction by identifying improvements in ankle angle on the affected side and the degree of asymmetry of both the affected and unaffected ankle angles [27]. However, there is limited information available regarding the kinematics of other lower limb joints and spatiotemporal gait asymmetry parameters after ankle dorsiflexion assistance during the swing phase of patients with foot drop using untethered fully portable pneumatic-powered AFOs (FP-PPAFOs).

This study aimed to investigate the effect of ankle dorsiflexion assistance on gait parameters in patients with foot drop. We hypothesized that ankle dorsiflexion assistance improves both kinematic and spatiotemporal gait asymmetries. For long-term deployment and high transmissibility of the pneumatic power from the AFO to the affected ankle, we improved both energy source modules with the AFO design. The hypothesis was validated by analyzing kinematic and spatiotemporal data from motion analysis.

## Method

### FP-PP AFO

**Fig. 1:**
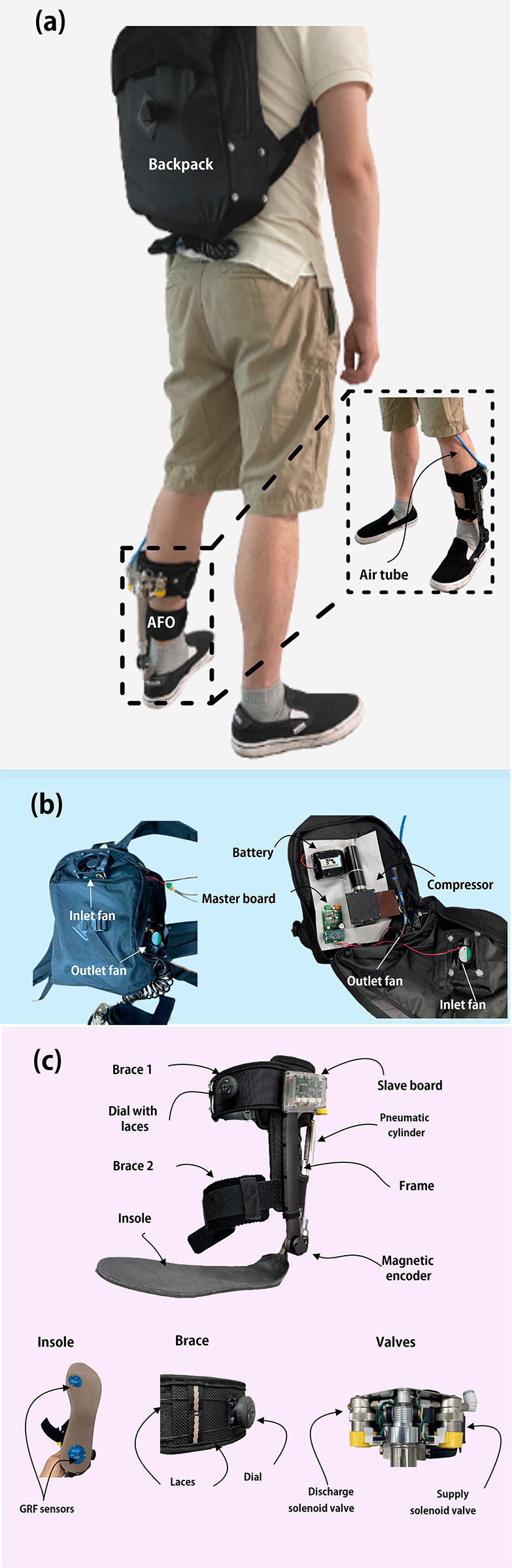
**System overview** **(a)** Picture of a person wearing an ankle-foot orthosis (AFO) with a backpack. **(b)** The backpack, as a pneumatic power source, comprises a compressor, battery, master board, and both inlet/outlet fans for heat dissipation. **(c)** The AFO consists of a pneumatic actuator, frame for fixation, and an insole. An air tube is connected between the compressor and pneumatic cylinder. Two ground reaction force (GRF) sensors are located on the toe and heel for gait phase detection. Calf braces placed in the top and the middle of the frame connect the AFO with the human leg and contribute to the high force transmissibility via a stable fixation of the static part of the pneumatic cylinder to the calf. The dial, including the laces in the brace, enables the adjustment of the fixation of the AFO frame to the calf. Supply and discharge solenoid valves are implemented for AFO control based on the GRF sensor signal

In this study, we used an upgraded AFO system that we had previously used [27, 29]. This system provides ankle dorsiflexion assistance during the swing phase; it consists of a backpack with a customized compressor that generates compressed air, an AFO unit, and an actuator that assists ankle dorsiflexion, connected to the foot and leg from the energy transmitted via the air tube (Fig. 1). The compressor continued to operate and provided compressed air to the AFO. A schematic of the AFO system control can be summarized in two stages. First, gait phase detection is performed using a total of two ground reaction force (GRF) sensors (I2A Systems, Republic of Korea) each located in the heel and toe of the insole. According to Fig. 1, it can be observed that the patient wore the AFO only on one ankle. Thus, the total number of the GRF used in the experiments is two sensors in a single ankle of the affected side. Next step, by controlling the supply and discharge valves (VQ100, SMC, Japan), the pneumatic cylinder (SD16N75-B, Taiyo, Japan) operates to assist the ankle dorsiflexion. During a single gait cycle, the compressed air generated by the compressor is stored in an air tube in the stance phase and transmitted to the pneumatic cylinder in the AFO during the swing phase. Based on previous studies [30, 31], the maximum torque for ankle dorsiflexion was 10 Nm. Murray et al. [33] reported that the average duration of the gait cycle ranges from 0.98 to 1.07 s. In addition, the stance phase occupies 60% of the gait cycle. Thus, compressed air from the compressor must be supplied to the air tube and stored during the stance phase. This air is then transmitted to a pneumatic cylinder during the swing phase for dorsiflexion assistance.

The output torque of the AFO is determined by the output force of the cylinder and moment arm of the rotary joint. Here, the output force of the cylinder is calculated as the integral sum of the flow rate over the swing period. The moment arm of each AFO used in this study is approximately the same. Further, we used the same cylinder for each patient with the same flow rate. However, the swing period times of each participant were different. Therefore, the output torques for each user were different. This contributes to a different output torque for each patient. A DC motor (RE40, Maxon Motor, Switzerland) with a two-stage planetary gear (GP 52 C, Maxon Motor, Switzerland) was used as the input for the compressor. Additionally, a Li-ion battery pack (33.6 V for 5.7 Ah, Powercraft, Republic of Korea) was selected. The pneumatic compressor provided compressed air at a pressure of 550 kPa and a volume flow rate of 1.5 mL/s, which produced a maximum assistance torque of approximately 9.3 Nm and charged the required air for the cylinder in the air tube within approximately 0.6 s.

Despite the noticeable effect in the performance of ankle kinematics improvement of the drop foot patient using our previous system [27, 29], certain practical issues in both the compressor and AFO limit the long-term use and stable force transmission in daily usage of the proposed system.

To improve the usability and reliability of our previous fully portable pneumatic-powered AFO, we improved the design in two ways: (1) implementation of the heat dissipation structure using inlet/outlet fans (SUNON, China) in the backpack comprising the compressor, and (2) brace design with a wire-fastening structure in two parts of the calf, as shown in Fig. 1. Following 1 h of continuous operation of the compressor, the maximum temperature of the backpack reached 40 °*C*, which was approximately 45 °*C* lower, compared to the system without heat dissipation. A dial with laces (BOA Fit System, USA) was implemented in the calf brace to increase the force transmissibility of the AFO by adjusting the fixation to the calf. A customized compressor employing a double-piston cylinder mechanism was used as the pneumatic power source, as introduced in our previous studies [26, 27]. The total weights of the backpack and AFO were approximately 2.9 and 0.45 kg, respectively. The system weight is known to highly contribute to the performance of the wearable robot. Compared to the previously reported AFOs in the literature [28], the proposed AFO is the lightest (0.45 kg). The lightest AFO reported in the previous study was PAM (pneumatic artificial muscle) of 0.67 kg. This results in a high torque to weight ratio of the AFO system (~ 3.3 Nm/kg). Furthermore, we verified that approximately 10 Nm of assistance torque could improve the gait asymmetry.

The continuous usage time of the compressor and AFO after the battery was fully charged was approximately 65 min. Insole sizes were used to accommodate users of various foot sizes.

### Control

**Fig. 2:**
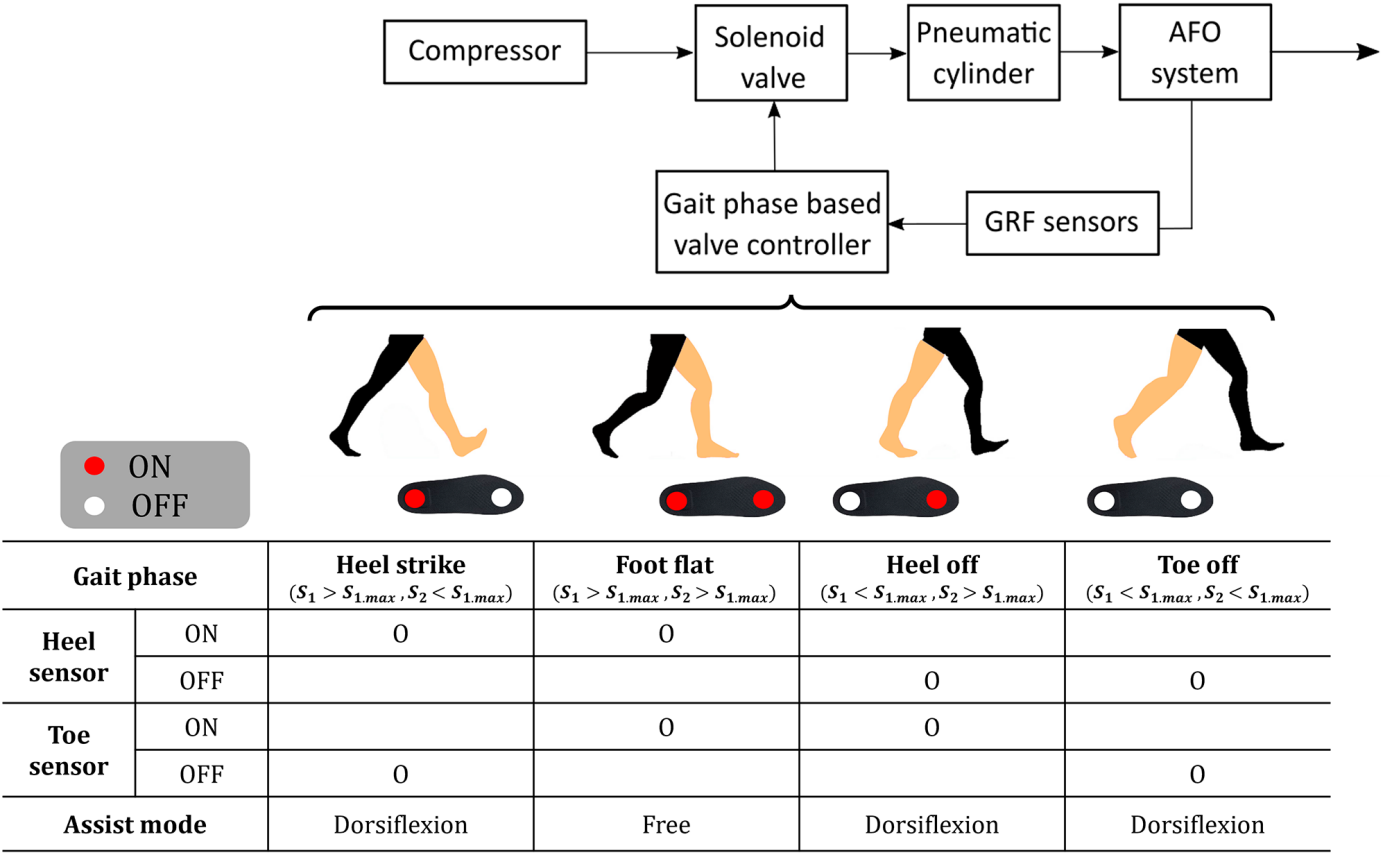
**Schematic of the proposed AFO control** The block diagram represents the ankle-foot orthosis (AFO) control strategy based on the gait-phase detection and valve control. We simplified the gait event with four different configurations: heel strike, foot flat, heel off, and toe-off in series (skin-colored leg). The process from the start of the heel strike to the end of the heel off is called the stance phase of the gait cycle. After toe off, the foot is off the ground, and the swing phase starts until the next heel strike event. To provide a control input of the patient’s gait phase to the AFO, we first designate the boundary value of each GRF sensor (${s}_{1.max}$: heel boundary value, and ${s}_{2.max}$: toe boundary value). The boundary value is determined as the sensor value (${s}_{1}$: heel value, and ${s}_{2}$: toe value) when each part of the insole (heel and toe) of each person touches the ground. This leads to the dorsiflexion assistance during the entire gait period except when the foot is flat

Figure 2 shows a detailed schematic of the proposed AFO system control. The compressed air generated by the compressor was consistently transmitted to the air tube independent of the gait phase. Controlling the solenoid valve determines the pneumatic power transmission from the compressor to the pneumatic cylinder or AFO system. A threshold-based gait phase-detection algorithm for the valve controller was implemented. First, the value indicated by each sensor when the heel and toe contacted the ground was set as the boundary value for each GRF sensor (${s}_{1.max}$ for the heel and ${s}_{2.max}$ for the toe). The purpose of our AFO system was to improve the gait of patients with foot drop. The simplest but most essential strategy was to prevent foot drop during the entire gait cycle. Based on this concept, the proposed AFO system assisted ankle dorsiflexion during the swing phase. We considered the heel strike as a late swing and the heel as an early swing, which indicated that our AFO control strategy focused on ankle dorsiflexion assistance during the swing phase (assist mode in the table in Fig. 2). This also implies that the swing phase starts with the toe-off and ends with the heel strike [29].

### Subjects

Ten patients with foot drop (Seven males and three females, mean age 54.8 $\pm$ 14.1 years) participated in the experiment. All participants signed an informed consent form prior to the experiments. All experimental protocols were approved by the Institutional Review Board of the Seoul National University Hospital (SNUH IRB No. 1506-141-683). Detailed demographics of the patients are presented in Table 1.

**Table 1:** **Patient demographics**

No.	Sex (M/F)	Age (y)	Affected side	Post disease time (y)	Lesion
P1	M	68	Rt	-	Chronic right sciatic neuropathy
P2	M	40	Rt	4	Chronic right L5 radiculopathy
P3	F	73	Lt	-	Moderate partial axonotmesis
P4	M	65	Lt	-	Sensorimotor polyneuropathy
P5	M	65	Lt	11	Poly radiculopathy
P6	F	35	Lt	-	Chronic nerve or muscle injury
P7	M	44	Lt	-	Left peroneal neuropathy
P8	M	65	Lt	8	Left L5 radiculopathy
P9	F	34	Rt	11	Right sciatic neuropathy
P10	M	59	Rt	18	Right common peroneal neuropathy

Several criteria were set to determine whether patients who participated after selection were suitable for the experiment. The criteria for testing the suitability of the patients in this experiment are specified below. First, we selected the patients with spasticity of Modified Ashworth Scale (MAS) grade below 2. Second, we conducted an experiment targeting people capable of walking independently, although it is worth noting that this does not imply they walk without problems; rather refers to patients who could walk despite facing challenges. Third, we selected the patients with hemiplegia. Lastly, we excluded patients with serious contractures which limit the wearing the AFO. We initially selected patients based on the five criteria mentioned earlier. Subsequently, patients who encountered problems were excluded from the study, and other patients were selected. Our investigation identified conditions such as partial axonotmesis, polyradiculopathy, and left or right sciatic neuropathy among the patients. By investigation of the disease, partial axonotmesis, polyradiculopathy, and left or right sciatic neuropathy were reported among patients.

### Data acquisition

**Fig. 3:**
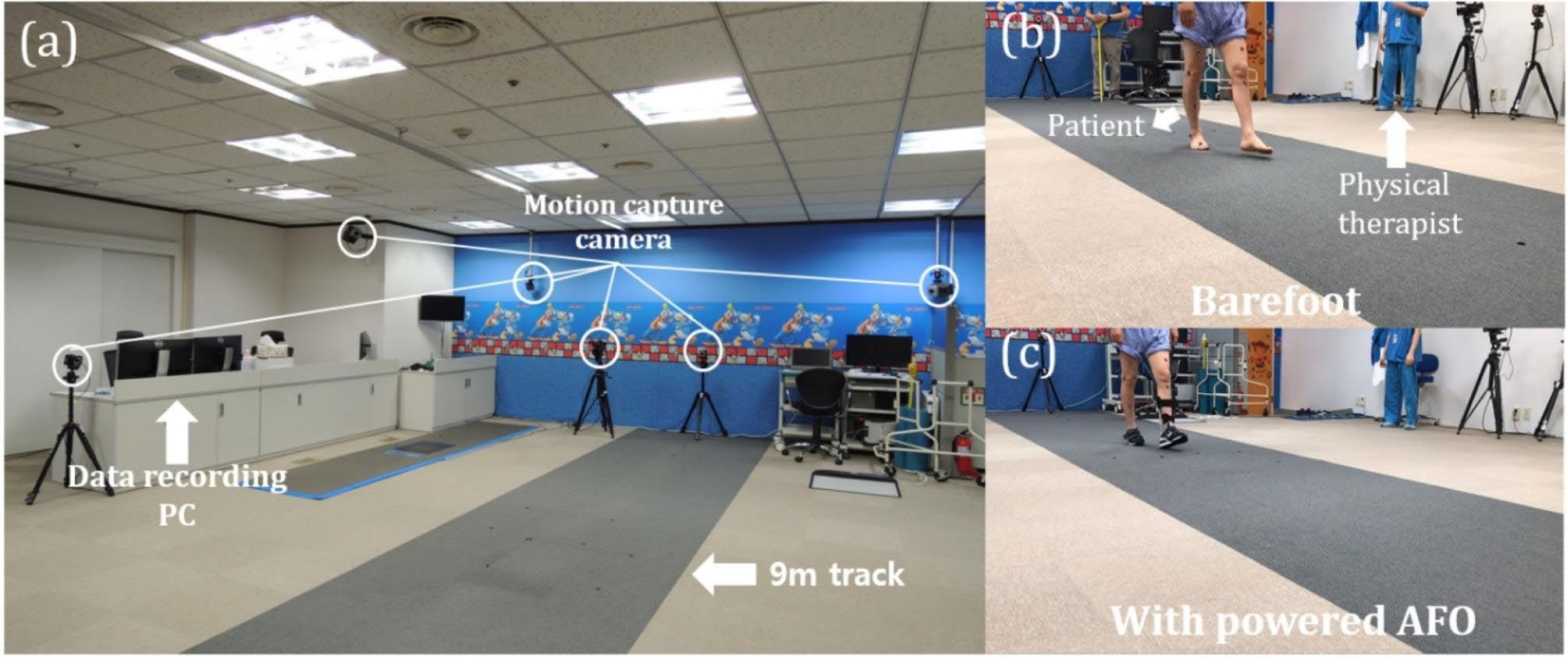
**Overview of the experiment** **(a)** Experimental setup consisting of a motion-capture camera, data-recording PC, and a 9 m track. Patients walked at a self-selected speed according to the physical therapist’s guideline. **(b)** The patient walking barefoot without any support. **(c)** The patient walking back and forth with a powered ankle-foot orthosis (AFO)

As shown in Fig. 3**(a)**, kinematic data were collected using 12 charge-coupled device cameras equipped with a three-dimensional optical motion capture system (Motion Analysis Co., Santa Rosa. CA), at a sampling rate of 120 Hz. Eight Eagle cameras were set at each octant position (45° intervals). Four Osprey cameras were placed at the back, front, and bilaterally. The translational accuracy was 0.5 mm root-mean-square, and the angular resolution was 0.3°. Cortex 8.1.0.2017 and Orthotrak 6.6.4 (Motion Analysis Co., Santa Rosa. CA) were used for real-time motion capture and post processing. The kinematic data were normalized to 101 data points over the gait cycle.

### Experimental protocol

Participants were asked to walk for 5 min to warm up. Subsequently, reflective markers were placed on the participants by an experienced operator using the David Hayes marker set. Baseline static data were obtained from a calibration trial with the foot positioned flat on the ground. Subsequently, subjects were asked to walk at their usual speed along a 9-m track. The patients walked under two different conditions: barefoot and in AFO-powered mode (Fig. 3**(b), (c)**). Ten laps of level-ground walking were performed for each condition, and patients rested for 5–10 min between each trial.

## Result

**Fig. 4:**
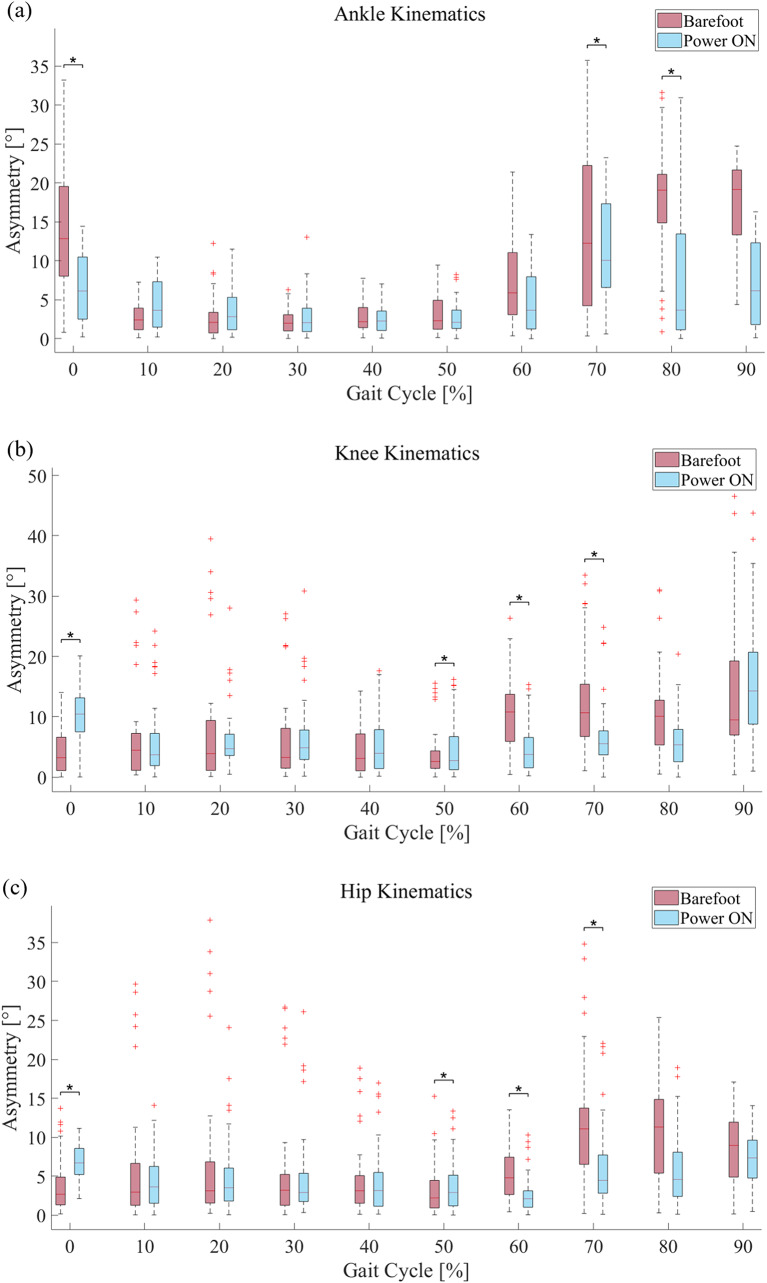
**Lower limb kinematic asymmetry** Statistic data of both barefoot mode and power on mode were described in each gait cycle. Statistically significant period was noted with asterisk. Kinematic asymmetry of the **(a)** ankle, **(b)** knee, and **(c)** hip

This section describes the lower-limb joint kinematics and spatiotemporal gait parameters of patients with foot drop during the gait cycle in the sagittal plane. First, the kinematics of the impaired lower-limb joints and kinematic asymmetry are discussed. Following the kinematic information, a spatiotemporal analysis, including the stride length, stance phase ratio, and swing velocity, is presented. We used a Wilcoxon signed-rank test method to identify the statistical significance between two conditions: barefoot walking and AFO ON of the lower limb joint asymmetry. Figure 4 describe the kinematic asymmetry during a gait cycle with statistical result. During the gait cycle, we compared the data distribution of each period of gait cycle from 0 to 90%. From the result, we identified a decreasing tendency in all three lower limbs asymmetry during early swing to mid swing phase (60–80% during the gait cycle). Moreover, in certain periods during the gait cycle, especially during the early stance (0%), and early swing to mid swing phase (60–80%), a statistically significant difference exists in all three lower limbs joints (Fig. 4. **(a), (b), (c)**). We noted the statistically significant period with asterisk to emphasize and discriminate with other periods.

### Ankle kinematics

**Fig. 5:**
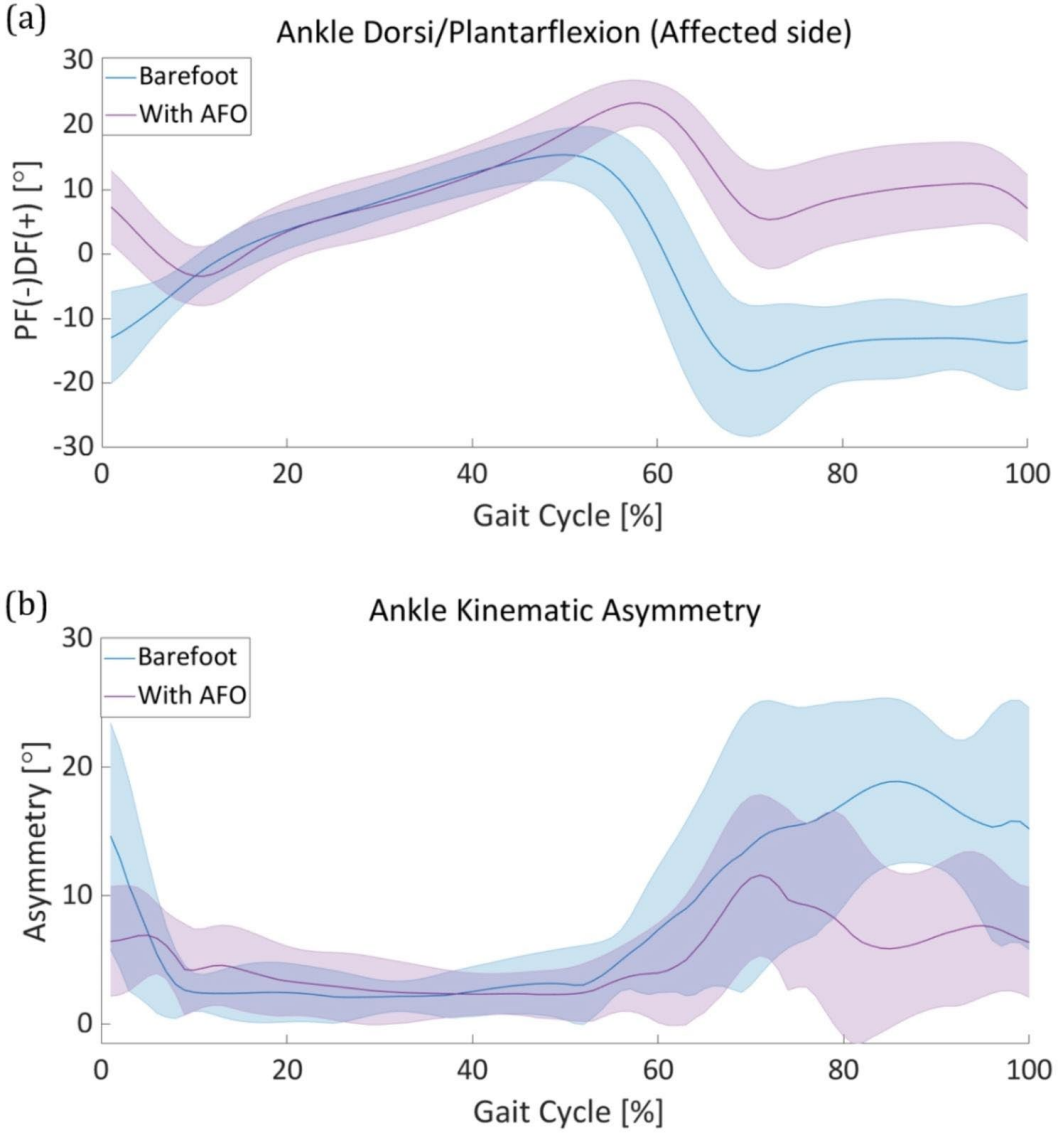
**Ankle kinematics analysis** The solid line and the shaded area represent the mean and standard deviation of the ankle asymmetry angle, respectively. **(a)** The mean and standard deviation of the ankle-joint angle of ten patients during the gait cycle in both barefoot (blue) and ankle-foot orthosis (AFO) (purple) modes. **(b)** The ankle kinematic asymmetry calculated as the difference between the affected and unaffected sides during the gait cycle in both barefoot (blue) and AFO (purple) modes

The AFO system directly affected the ankle joint kinematics. Figure 5**(a)** shows the ankle-joint angle of the affected side in both the bare-footed and AFO-ON modes. The ankle dorsiflexion angle of the affected side improved by approximately 20° during the late swing period (95–100%) of the gait cycle compared to the barefoot condition. An increase in ankle angle during the corresponding period is the most important index for improving the quality of gait in foot drop patients through active ankle assistance during the gait cycle. In the early stance phase (0–20% in the gait cycle), the ankle trajectory showed a natural plantar flexion from the increased ankle angle with dorsiflexion assistance. During the swing phase, an overall improvement in the ankle-joint angle was observed in the AFO ON mode, compared to barefoot walking. The average ankle-joint angle did not fall below 0° and remained at approximately 0° at the end of the swing phase. Over the entire gait cycle, the ankle-joint angle graph showed 7–8% delay during the AFO-ON mode, compared to the barefoot mode (Fig. 4**(a)** 50–60% in the gait cycle).

The kinematic asymmetry of the ankle joint is evaluated by calculating the difference between the average ankle-joint angles in the barefoot and AFO-ON modes (Fig. 4 (b)). The kinematic asymmetry of the other lower-limb joints is also derived as in Eq. (1);

1 $${ASS}_{kinematic}={AF}_{mean}-{UAF}_{mean}$$

where ${ASS}_{kinematic}$ is the kinematic asymmetry of the lower limb joint, ${AF}_{mean}$ is the average joint angle on the affected side, and ${UAF}_{mean}$ is the average joint angle on the unaffected side.

### Knee kinematics

**Fig. 6:**
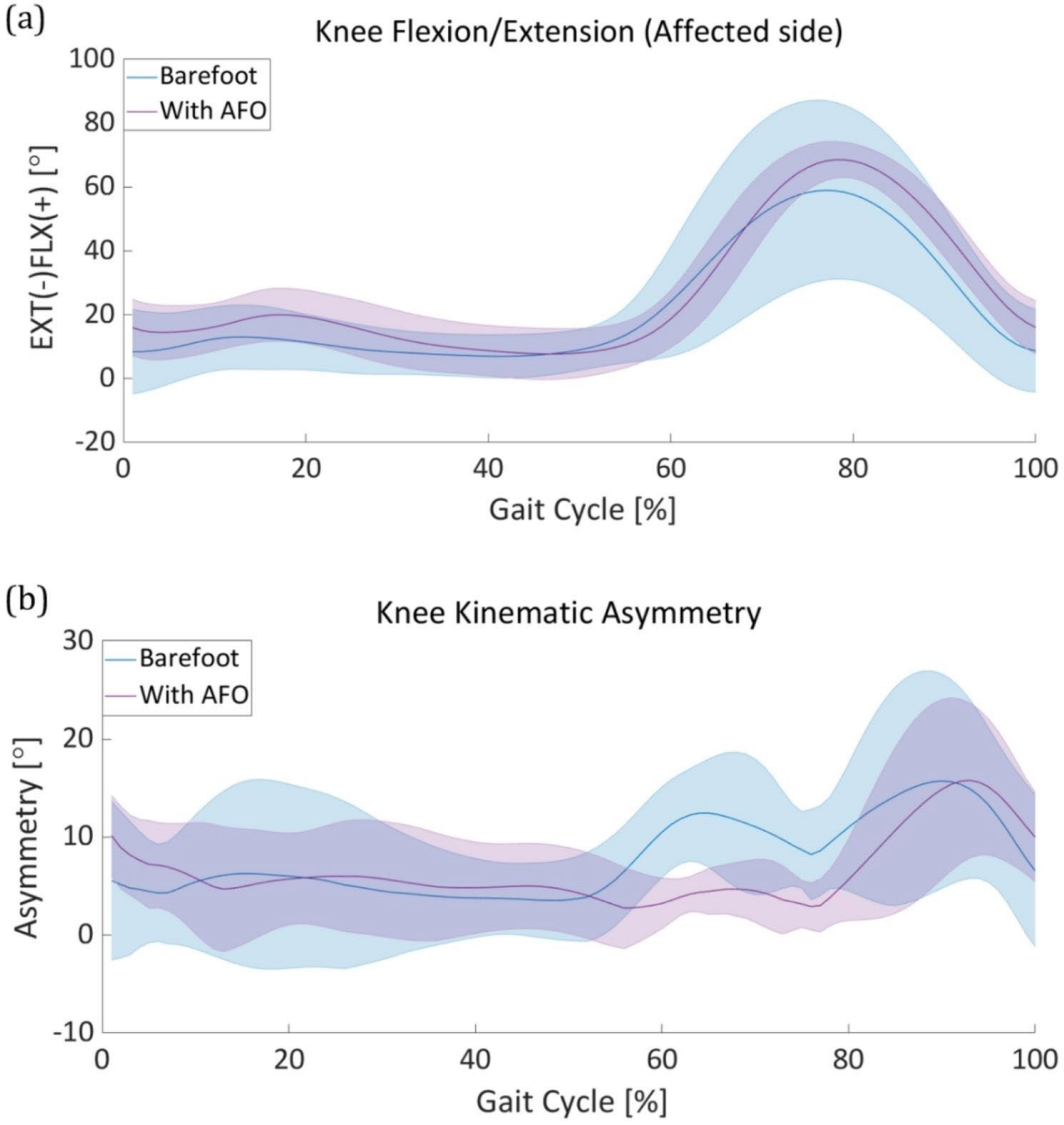
**Knee kinematics analysis** The solid line and the shaded area represent the mean and standard deviation of the knee asymmetry angle respectively. **(a)** The mean and standard deviation of the knee joint angle of ten patients during the gait cycle of both barefooted (blue) and with AFO (purple). **(b)** The knee kinematic asymmetry calculated as the difference between the affected side and the unaffected side during the gait cycle of both barefooted (blue) and with AFO (purple)

Following the improvements in ankle kinematics after using the AFO, we expected improvements in other lower-limb joint motions. Figure 6**(a)** shows the knee-joint angle in both bare foot and AFO-ON modes. Compared to the barefoot mode, the maximum knee flexion angle increased by approximately 15° after the AFO assistance was used (Fig. 6**(a)**). Furthermore, in barefoot walking, the standard deviation of the knee-joint angle among the patients showed abnormally high trends during the swing phase (60–100% in the gait cycle, Fig. 6**(b)**). However, it was discovered that ankle dorsiflexion assistance during this period significantly reduced the standard deviation of the knee angle particularly during the swing phase.

### Hip kinematics

**Fig. 7:**
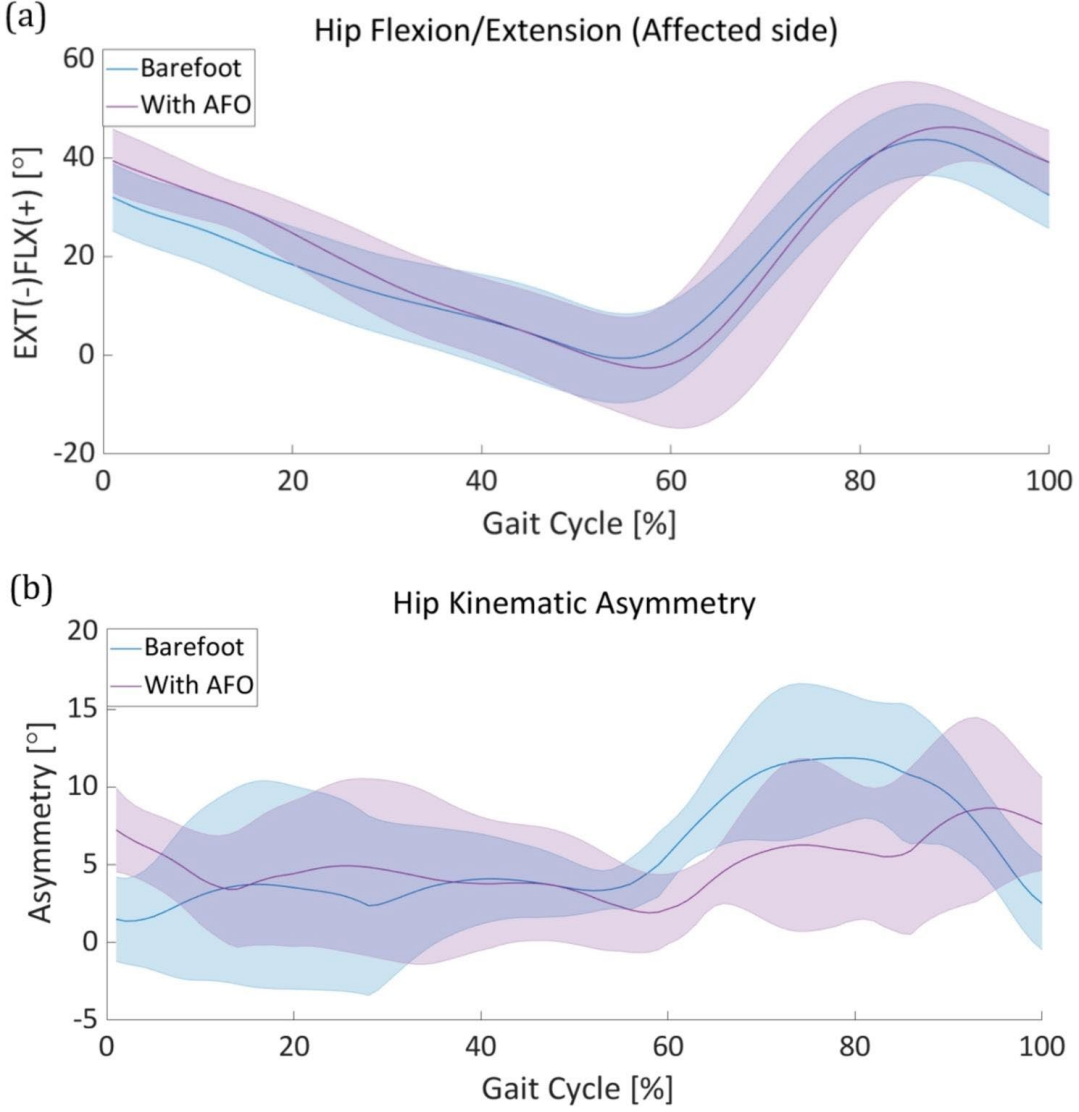
**Hip kinematics analysis** The solid line and the shaded area represent the mean and standard deviation of the hip asymmetry angle, respectively. **(a)** The mean and standard deviation of the hip-joint angle of ten patients during the gait cycle in both barefoot (blue) and ankle-foot orthosis (AFO) (purple) modes. **(b)** The hip kinematic asymmetry calculated as the difference between the affected and unaffected sides during the gait cycle in both barefoot (blue) and AFO (purple) modes

Foot drop patients typically perform compensatory motions in the hip joint to prevent toe drag owing to their low ankle dorsiflexion ability. These patients excessively flex the hip joint on the affected side, leading to an unbalanced gait pattern. In this study, we focused on kinematic data of the sagittal plane of hip joint motion. First, we checked the hip joint angle in both the barefoot and AFO-ON modes. Unlike other lower extremity joints, the average hip-joint angle data of the patients before and after assistance did not show a significant difference (Fig. 7**(a)**). Additionally, the standard deviation of the hip-joint angle increased slightly after using the FP-PPAFO, compared to the barefoot walking condition (Fig. 7**(a)**).

We then investigated the asymmetry of hip motion by calculating the difference between the hip angles of the affected and unaffected sides using Eq. (1), similar to other lower limb joints (Fig. 7**(b)**).

### Spatiotemporal analysis

**Fig. 8:**
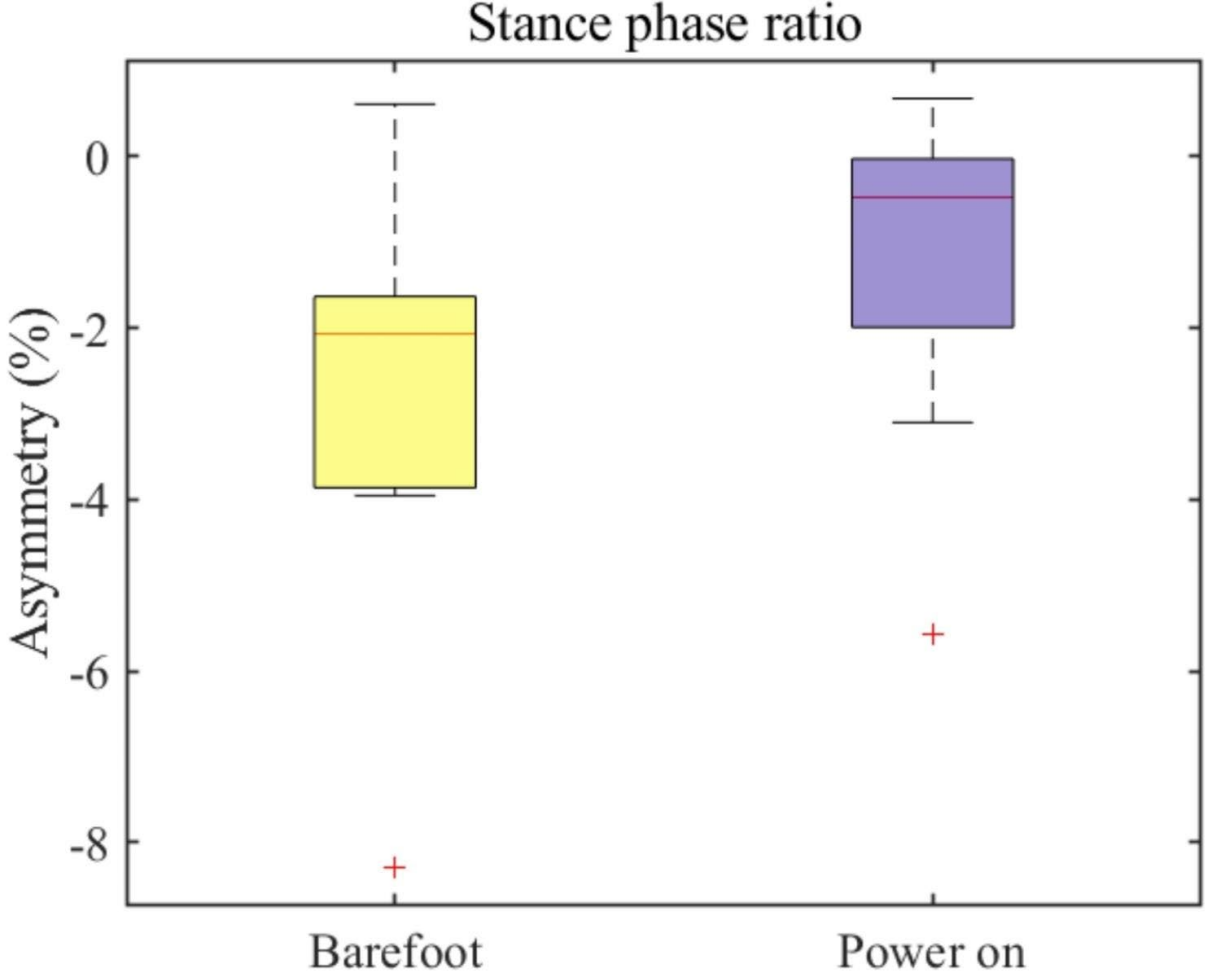
**The asymmetry of the stance phase ratio** Box chart of the stance phase ratio asymmetry in both barefooted (yellow) and power-on (purple) modes

**Fig. 9:**
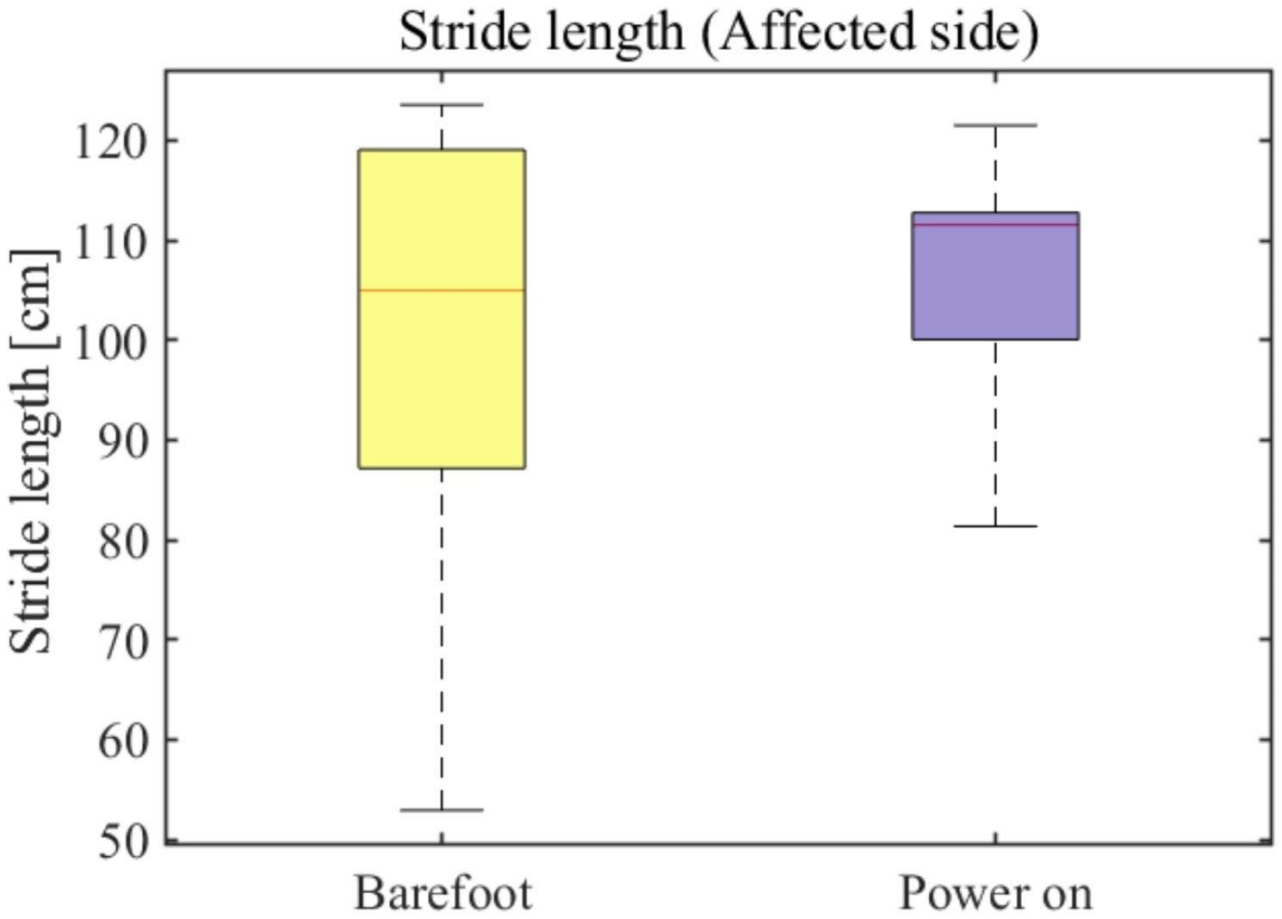
**Stride length of the affected side** Box chart of the stride length at the affected side in both barefoot and power-on modes

**Fig. 10:**
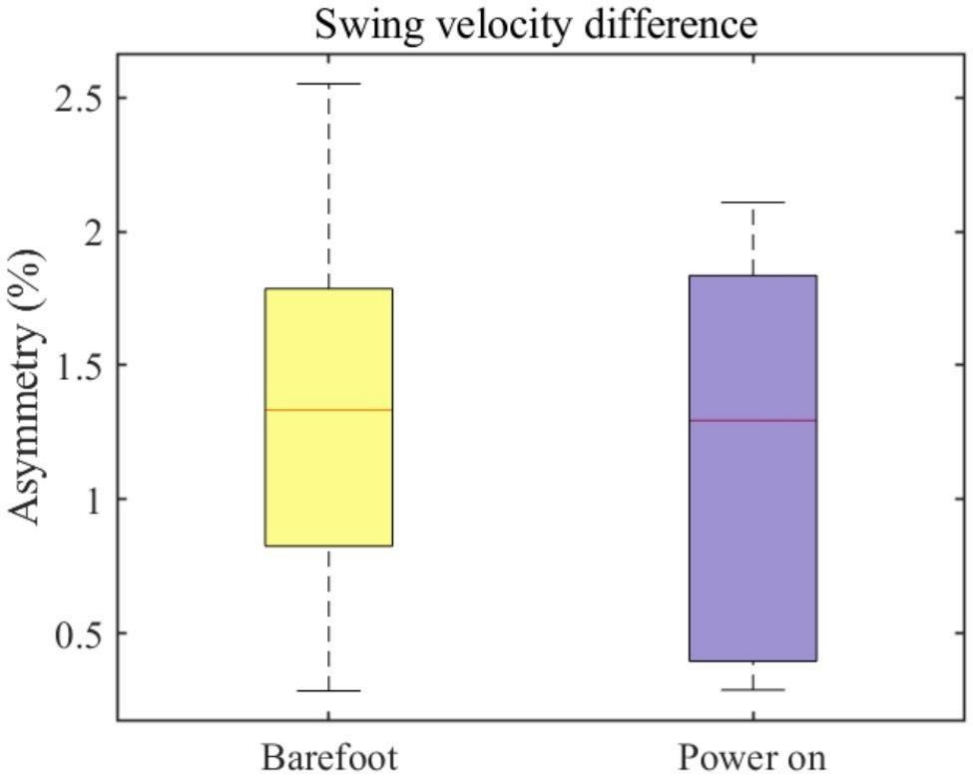
**Asymmetry of the swing velocity difference** Box chart of the swing velocity asymmetry in both barefoot (yellow) and power-on (purple) modes

In addition to kinematic information, a spatiotemporal analysis provided comprehensive insights into gait patterns. This study focused on three spatiotemporal parameters: stance phase-ratio asymmetry during the gait cycle, stride length, and swing velocity asymmetry. An appropriate stance phase ratio during the gait cycle enables a natural transition to the swing phase and plays an essential role in the stable movement of the human body. In this context, we evaluated stance phase asymmetry in the ten foot drop patients and summarized it using a box chart (Fig. 8). We calculated the asymmetry of the stance phase ratio using Eq. (2)

2 $${AS}_{stance}=\frac{AF-UAF}{\text{m}\text{a}\text{x}(AF,UAF)}\bullet 100$$

where ${AS}_{stance}$ is the stance phase-asymmetry ration, $AF$ is the stance phase ratio of the affected side, and UAF is the stance phase ratio of the unaffected side. After the dorsiflexion assistance, the overall average asymmetry of the stance phase ratio decreased by approximately 70%, from 2.1% in the barefoot mode to 0.7% in the AFO power-on mode (see red line in Fig. 8). Moreover, the maximum asymmetry of the stance phase ratio was approximately 3% (minimum value in the power-on mode in Fig. 8), that is, a decrease of approximately 25% of the maximum asymmetry of the stance phase ratio during the barefoot condition, which was approximately 4% (minimum value in the barefoot mode in Fig. 8). The stride length of the affected side is shown in Fig. 9. The average stride length of the affected side increased by approximately 9 cm, from 104 to 113 cm, after using the AFO. Furthermore, a noticeable improvement in the stride length has been shown in the lower boundary (0.25 quantile of the stride length) from 86 to 102 cm. This indicates that the 0.25 quantile of the mean stride length increased by approximately 30% after the ankle dorsiflexion assistance. The difference in the swing velocity between the affected and unaffected sides was calculated to provide a temporal index of asymmetry. We derived the swing velocity using Eq. (3).

3 $${AS}_{sw\_v}=\frac{{AF}_{sw\_v}-{UAF}_{sw\_v}}{\text{m}\text{a}\text{x}({AF}_{sw\_v},{UAF}_{sw\_v})}\bullet 100$$,

where ${AS}_{sw\_v}$ denotes the difference in the swing velocity, ${AF}_{sw\_v}$ is the swing velocity on the affected side, and ${UAF}_{sw\_v}$ is that on the unaffected side. The detailed results of the asymmetry of the swing velocity difference between the affected and unaffected sides are shown in Fig. 10. The average swing velocity asymmetry on both sides was nearly identical, approximately 1.25%. However, the 0.25 quantile of the swing velocity difference ${ASS}_{sw\_v}$ of the power-on mode was approximately 0.4%, which is comparable to that of healthy people [33]. Moreover, the maximum value of the swing velocity difference in power-on mode was approximately 2%, which was 20% lower than the difference of 2.5% in barefoot mode.

Similar to the kinematic asymmetry analysis, we also executed a statistical test to validate the acquired spatiotemporal parameter dataset. The swing velocity asymmetry and the stride length of the affected side showed no significant statistical difference between barefoot walking and AFO ON mode walking (**p = 0.870, p = 0.278** for each case respectively). However, for the stance phase ratio, a statistical significance exists between the barefoot walking and AFO ON mode walking (**p** < 0.001).

## Discussion

Previous studies reported multiple AFO designs and control strategies implemented for drop foot prevention, correction, and rehabilitation [27, 34–42]. Gait asymmetry is an important issue among foot drop patients. As mentioned in the earlier chapter, the lack of ankle dorsiflexion muscles in neuromuscular disorders affects the other lower limb joints and leads to compensatory motions such as the hip hike. However, little is known about a comprehensive analysis of gait asymmetry after AFO use in patients with foot drop.

In this study, we investigated the efficacy of a fully portable compressor-based pneumatic-powered unilateral ankle dorsiflexion assistance in improving gait asymmetry in patients with foot drop based on kinematic and spatiotemporal analyses. Event-based valve control was used to operate the pneumatic AFO for ankle dorsiflexion assistance during the swing phase (starting from heel-off to the end of the toe-off, and the end of the toe-off to the heel strike). This restrained the excessive foot drop throughout the gait cycle. The average peak dorsiflexion angle during the swing phase showed approximately 20° improvements after using the AFO. This implies the prevention of foot drop during the swing phase and contributes to the natural transition from the swing to the stance phase. After reaching the maximum dorsiflexion position, the ankle plantarflexion located the ankle position below 0° for natural gait. However, the restrained ankle angle for preventing the foot drop during the swing phase resulted in a lack of plantar flexion (60–75% in the gait cycle (Fig. 4**(b))**, compared to healthy people. The performance of the valve, particularly the flow rate, determines the pressure profile that contributes to the assistance. We expected an improvement in ankle plantar flexion during the swing phase by increasing the discharge valve flow rate in the AFO. The asymmetry of the ankle-joint angle significantly improved by more than 50% throughout the swing phase. We believe that the increased ankle-joint angle on the affected side may have resulted in the improvement of ankle joint asymmetry. For the knee joint, the average flexion angle increased during the gait cycle (Fig. 6. **(a)**) and the standard deviation of the knee angle noticeably decreased to during the swing phase (60–100% in the gait cycle, Fig. 6**(a)**). The improved ankle dorsiflexion angle during the swing phase may have mitigated the importance to compensate for the foot drop by excessively bending the knee. For these reasons, a decreased knee asymmetry angle was confirmed throughout the entire gait cycle, particularly during the early swing phase to mid swing phase (55–80% in the gait cycle, Fig. 6**(b)**). Similarly, the hip asymmetry angle represents a significantly decreasing trend after using the AFO from the early swing phase to the late swing phase (55–90% in the gait cycle (Fig. 7**(b)**). Based on the results of the improved average lower-limb joint angle asymmetry, we verified that ankle dorsiflexion assistance during the swing phase contributes to alleviating the kinematic asymmetry of patients with foot drop during the gait cycle. The average values ​​of joint asymmetry angles were commonly improved in all three joints of the lower extremities (ankle, knee and hip). Conversely, however, the standard deviation improved in the ankle and knee joints, but showed no noticeable change in the hip joint and rather showed a slight increase in asymmetry. The degree of improvement of the average asymmetry angle was the highest in the order of the ankle joint, knee joint, and hip joint, with 3.2°, 1.2°, and 0.7°, respectively, and the ratios were 38%, 15%, and 12.5%, respectively.

From this result, we conclude that the proposed system and control strategy impact the asymmetry correction of the ankle joint primarily. In addition to kinematic analysis, we focused on spatiotemporal analysis. Based on the stance phase ratio of the unaffected and affected sides, we evaluated the asymmetry of the stance phase ratio using the difference between the two measures. During the stance phase, the lower limbs supported the body and extended to perform the required push-off [43]. The stance phase ratio varied depending on gait speed [44], and speed is highly correlated with the extent of abnormalities, such as hemiplegic gait patterns [44]. Based on this, the reduced asymmetric stance phase ratio indicates the improved gait ability of the patient with foot drop after receiving ankle dorsiflexion assistance from with our developed AFO system during the swing phase. In the same context, the decreased asymmetry of the swing velocity also indicates a refined gait ability from assistance. After the corrected kinematic asymmetry from the ankle dorsiflexion assistance, it was confirmed that the average stride length of a patient with foot drop increased by approximately 10%, compared to the barefoot mode. From this result, we conclude that the proposed assistance strategy led to improvements in the spatial parameters of the gait cycle.

Although the experiment was conducted under self-selected walking conditions, the question of whether ankle dorsiflexion assistance in patients with foot drop is effective at fast walking speed remains unresolved. Future studies might include the experimental results in multiple speed conditions and validate the performance of the proposed AFO system. Especially for fast walking conditions, we expect a longer stride length compared to that of the self-selected walking speed. Furthermore, this might explain the effect of ankle dorsiflexion assistance during the swing phase more dramatically and show the feasibility of the developed AFO in practical usage in terms of multiple walking speeds.

The backpack of 2.9 kg weight might be an obstacle in long-time usage in real world. However, there were no reports regarding the inconvenience from the patients from our study result. Although the user experience did not show a high level of inconvenience among the users, decreasing the backpack weight could improve the user performance and convenience. Therefore, we plan to minimize the portable pneumatic compressor and other components and decrease the backpack weight compared to the current version for better usability. In our previous study [27, 29], we only monitored the corrected angle and the asymmetry angle of the ankle between unaffected and affected sides and focused on the performance of the portable AFO system itself.

Compared to our previous studies, we focused on the gait asymmetry of all three joints by calculating the kinematics and spatiotemporal parameters. In addition, the total number of patients participating in these experiments increased from 4 to 10, which enriched the statistical analysis. Moreover, the investigation of the muscle activity before and after the dorsiflexion assistance might provide a better understanding of the gait asymmetry improvements. Thalman et al. [45] reported a soft pneumatic AFO with a maximum torque of 1.2 Nm that reduced the muscle activity by 13.3% used during the ankle dorsiflexion. We expect that our suggested system will enhance the economy of the muscle activation required for ankle dorsiflexion better than that previously reported [45] based on higher torque capacity for multiple environments of walking.

## Conclusion

The results of this study prove that ankle dorsiflexion assistance during the swing phase improves gait asymmetry in patients with foot drop. We upgraded our previously developed fully portable pneumatic-powered AFO system by preventing overheating and implementing a customizable calf brace to enhance the force transmissibility of the AFO. Using the developed AFO system, we evaluated the kinematic change and asymmetry of both lower-limb joint kinematics and spatiotemporal parameters including the stance phase ratio, stride length, and swing velocity. The experimental results demonstrate the promising efficacy of the developed AFO system for lower-limb joint kinematic improvement and asymmetry reduction of spatiotemporal gait parameters.

In the future, we will evaluate the proposed system in unstructured environments for a real-world implementation. These environments include an obstacle-rich site, a terrain with an irregular slope, etc. Additionally, we will examine the muscle activity of the lower limbs during the gait cycle of patients with foot drop. Through muscle activity information, changes in the musculoskeletal system that are difficult to interpret with physical values during the gait cycle after using an AFO can be discovered. We expect the acquired information could provide insights into the rehabilitation field.

Thalman et al. [45] developed a socket-like soft actuator made of fabric for foot drop assistance. The developed system also assists dorsiflexion during the swing phase. However, the research focused exclusively on healthy people and did not involve patients with foot drop. In addition, the result of this study only focused on the reduction of the metabolic cost, not the kinematic and spatiotemporal improvements from the ankle dorsiflexion assistance. The maximum torque of 1.2 Nm also limits the usage of the AFO for multiple participants with foot drop. To the best of our knowledge, this study is the first to investigate both the kinematic and spatiotemporal gait asymmetry before and after using AFO for ankle dorsiflexion assistance during the swing phase of the patient with foot drop. However, this study only investigated the immediate effect after ankle dorsiflexion assistance during the swing phase. To validate the efficacy of the proposed system from a rehabilitation perspective, tracking the long-term change in the gait asymmetry of the kinematics and spatiotemporal parameters is required.

## Data Availability

The datasets may be available from the corresponding authors on reasonable request.
